# Amplification of temperature extremes in Arabian Peninsula under warmer worlds

**DOI:** 10.1038/s41598-024-67514-8

**Published:** 2024-07-18

**Authors:** Buri Vinodhkumar, Safi Ullah, T. V. Lakshmi Kumar, Sami G. Al-Ghamdi

**Affiliations:** 1 Biological and Environmental Science and Engineering Division, Environmental Science and Engineering Program, King Abdullah University of Science and Technology (KAUST), 23955-6900 Thuwal, Saudi Arabia; 2 KAUST Climate and Livability Initiative, King Abdullah University of Science and Technology (KAUST), 23955-6900 Thuwal, Saudi Arabia; 3 Department of Earth and Atmospheric Sciences, National Institute of Technology Rourkela, Rourkela, 769008 India; 4 School of Environmental Sciences, Jawaharlal Nehru University, New Delhi, 110067 India

**Keywords:** Temperature extremes, NEX-GDDP-CMIP6, SSPs, Global warming levels, Arabian Peninsula, Climate sciences, Natural hazards

## Abstract

The Paris Agreement and the Special Report on Global Warming of 1.5 °C from the Intergovernmental Panel on Climate Change (IPCC) highlighted the potential risks of climate change across different global warming levels (GWLs). The increasing occurrence of extreme high-temperature events is linked to a warmer climate that is particularly prevalent in the Arabian Peninsula (AP). This study investigates future changes in temperatures and related extremes over AP, under four GWLs, such as 1.5 °C, 2.0 °C, 3.0 °C, and 4.0 °C, with three different Shared Socioeconomic Pathways (SSPs: SSP1-2.6, SSP2-4.5, and SSP5-8.5). The study uses high-resolution datasets of 27 models from the NASA Earth Exchange Global Daily Downscaled Projections of the Coupled Model Intercomparison Project Phase 6 (NEX-GDDP-CMIP6). The results showed that the NEX-GDDP-CMIP6 individual models and their multi-model means reasonably captured the extreme temperature events. The summer maximum and winter minimum temperatures are projected to increase by 0.11–0.67 °C and 0.09–0.70 °C per decade under the selected SSPs. Likewise, the projected temperature extremes exhibit significant warming with varying degrees across the GWLs under the selected SSPs. The warm temperature extremes are projected to increase, while the cold extremes are projected to decrease under all GWLs and the selected SSPs. Overall, the findings provide a comprehensive assessment of temperature changes over AP in response to global warming, which can be helpful in the development of climate adaptation and mitigation strategies.

## Introduction

The global surface temperature has risen by 0.99 °C (0.84 °C–1.10 °C) from the period of 1850–1900 to the first two decades of the twenty-first century^1^. Due to the recent and projected increase in temperature with catastrophic impacts, the Paris Agreement, adopted by 196 parties across the globe at the Conference of the Parties (COP21) in 2015, introduced a global warming target, aiming to constrain the increase to well below 2 °C and preferably limiting it to 1.5 °C compared to pre-industrial levels^2^. The Intergovernmental Panel on Climate Change (IPCC) Special Report on global warming of 1.5 °C (SR1.5), issued in 2018, underscores that the mean global surface temperature has already experienced an increase of approximately 1.0 °C and is expected to reach the 1.5 °C mark sometime between 2030 and 2052 if the current rate of warming persists^2^. This prominent increase in global temperature will heighten the occurrence of climate extremes^3^. In recent times, there have been more frequent and intense temperature extremes associated with global warming across various regions worldwide^4,5^. Recently, Schleussner et al.^6^ analyzed the global and subcontinental-scale shifts in various climate extremes and associated impacts under 1.5 °C and 2 °C warming levels and reported significant distinctions between global warming scenarios of 1.5 °C and 2.0 °C. Given the catastrophic impacts of additional 0.5 °C warming, the robust and comprehensive future climate projections can be pivotal in delivering trustworthy insights regarding climate mitigation and adaptation. The outcomes of such projections will enable participating countries to tackle anthropogenic climate change and its adverse consequences effectively.

Like other parts of the world, the Arabian Peninsula (AP) has also experienced an increase in extreme temperature events^7–9^. This region exhibits significant variations in climate, geography, and social settings, which account for the amplification of temperatures and their extremes^10–12^. AP, characterized by its arid climate, limited freshwater resources, and extreme temperatures, is highly vulnerable to the impacts of climate change. Water scarcity presents a significant challenge in the AP, which is further exacerbated by the high energy costs of desalination. The region experiences extreme temperatures, with summer temperatures often exceeding 40 °C and sometimes reaching up to 50 °C, posing health risks and increasing the demand for energy for cooling. Sandstorms and dust storms, worsened by droughts and climate change, impact air quality and respiratory health. Additionally, rising sea levels threaten coastal communities and infrastructure. Addressing these challenges requires coordinated efforts to enhance resilience and reduce greenhouse gas emissions both at regional and global scales.

The recent increase in temperature is undeniable, and the impacts of climate change are not evenly distributed across different regions of the world^1^. The temperature changes across eleven subdomains in Africa and the Middle East indicate that the AP is projected to experience a faster rate of warming (0.83 °C per decade) than other subdomains under the Representative Concentration Pathway (RCP) 8.5 scenario. Additionally, AP is expected to have higher variability compared to other subdomains of the Africa and Middle East regions^13,14^. It is projected that the frequency and duration of heatwaves will significantly increase, accompanied by an increase in extremely hot days and a decrease in cold nights in the AP region^13,15,16^. Some of the Coupled Model Intercomparison Project Phase 5 (CMIP5) model-based studies reported a persistent rise in the annual mean surface air temperature over the AP during the twenty-first century^11,17^. Despite high vulnerability to future climate change, very few studies have used CMIP6 models to provide a detailed description of the AP's future changes in temperature and its extremes. Recently, Almazroui et al.^10^ examined the prospective alterations in temperature across the AP region using CMIP6 models and projected a significant increase in temperature by the end of the twenty-first century under various Shared Socioeconomic Pathways (SSP) scenarios. However, their findings are limited to temperature changes only and are mostly based on raw model outputs, which are not bias-corrected and statistically downscaled.

Generally, the CMIP6 models have a coarse spatial resolution that often exceeds 100 km, making them unsuitable for various applications, such as risk assessment, adaptation strategies, and localized decision-making processes. However, the application of downscaling and bias-correction techniques significantly improves the spatial resolutions that help us in the comprehension of local-scale climate change patterns^18,19^. This can be achieved through the most recent simulations from the NASA Earth Exchange Global Daily Downscaled Projections of the CMIP6 (NEX-GDDP-CMIP6) models, which feature higher resolution, enhanced reliability, and improved physics^20^. The NEX-GDDP-CMIP6 models have high-resolution, statistically downscaled, and bias-corrected outputs, which are highly recommended for robust assessment of climate change risks at local and/or regional scales^21–23^. Recent studies have also used the new iteration of NEX-GDDP-CMIP6 datasets to assess changes in climate extremes in different parts of the world^21–27^.

Given the significance of NEX-GDDP-CMIP6 data, this study investigates the relative changes in temperature extremes over AP for the four different global warming levels (GWLs), including 1.5 °C, 2 °C, 3 °C, and 4 °C under SSP1-2.6, SSP2-4.5, and SSP5-8.5 scenarios using 27 models from the NEX-GDDP-CMIP6 dataset. The AP region has undergone significant warming, characterized by frequent and intense heat extremes, which are expected to persist in the future, potentially leading to more severe consequences^28–30^. Our objective is to enhance our comprehension of the effects of climate change under the global warming goals outlined in the Paris Agreement (COP21), explicitly focusing on 1.5 °C, 2 °C, 3 °C, and 4 °C of GWLs. To the best of our knowledge, none of the studies assess future changes in temperatures and their extremes to understand the impact of climate change over the AP region in various GWLs and SSPs using high-resolution, bias-corrected, and statistically downscaled NEX-GDDP-CMIP6 datasets. Nonetheless, this research will contribute to advancing climate change mitigation strategies and informing policy development for the AP, considering the Paris Agreement.

## Data and methodology

This study used the daily maximum temperature (T_max_) and minimum temperature (T_min_) outputs from 27 bias-corrected models under the SSP1-2.6, SSP2-4.5, and SSP5-8.5 scenarios (Table 1), which are prepared under the NEX-GDDP data from CMIP6 global climate model outputs (https://www.nccs.nasa.gov/services/data-collections/land-based-products/nex-gddp-cmip6). The bias correction spatial disaggregation (BCSD) algorithm is used to produce a high-resolution (0.25° × 0.25°) bias-corrected and downscaled dataset. The BCSD method represents a trend-sustaining statistical downscaling technique and is widely used in multiple meteorological studies^19,31,32^. The datasets span from the historical period from 1951–2014 and future projections (2015–2100) for SSP1-2.6, SSP2-4.5, and SSP5-8.5 scenarios^20^. In addition, the ERA5 reanalysis temperature dataset was obtained from the European Centre for Medium-Range Weather Forecasts (ECMWF) fifth-generation atmospheric reanalysis with a spatial resolution of 0.25° × 0.25°^33^. The ERA5 dataset is used as a reference to evaluate the performance of selected models in simulating the historical temperatures. The IPCC Sixth Assessment Report (AR6) recommended the period 1995–2014 as the reference period for calculating changes in climate extreme indices^1^. For calculating future projections, using this reference period with relatively stable climate conditions may provide more accurate projections in a global warming world. We used the multi-model mean (MMM) to project future changes in temperature extremes under different GWLs and SSPs. This approach helps to reduce the intermodal uncertainties and biases, originating from the individual models^23^. We selected eight extreme temperature indices, among which seven indices were from the recommended list of the Expert Team on Climate Change Detection and Indices (ETCCDI) by the World Meteorological Organization (WMO) (Table 2). Following Odnoletkova and Patzek's approach^7^, we have added one new extreme temperature index, namely hot days (HDs). The HDs index is defined as “the annual count of days when the daily T_max_ reaches 37.8 °C (100°F)”. Recent studies state that the absolute values-based indices, i.e., HDs (T_max_ > 37.8 °C) and tropical nights (TR: T_min_ > 20 °C) are relatively more representative of the AP's extreme climatic features and provide more meaningful information than the ones recommended by the ETCCDI^7,34^. Moreover, the significance of the projected spatial changes in temperatures and their extremes under the selected GWLs is determined using Student's t-test at a 99% confidence level.

**Table 1:** Description of the selected NEX-GDDP-CMIP6 models for this study^20^.

Model name	Institution/Country
ACCESS-CM2	CSIRO-ARCCSS/Australia
ACCESS-ESM1-5	CSIRO/Australia
BCC-CSM2-MR	BCC/China
CanESM5	CCCma/Canada
CMCC-ESM2	CMCC/Italy
CNRM-CM6-1	CNRM-CERFACS/France
CNRM-ESM2-1	CNRM-CERFACS/France
EC-Earth3	EC-Earth-Consortium/EC-Earth consortium
EC-Earth3-Veg-LR	EC-Earth-Consortium/EC-Earth consortium
FGOALS-g3	CAS/China
GFDL-ESM4	NOAA-GFDL/USA
GISS-E2-1-G	NASA-GISS/USA
HadGEM3-GC31-LL	MOHC, NERC/UK
INM-CM4-8	INM/Russia
INM-CM5-0	INM/Russia
IPSL-CM6A-LR	IPSL/France
KACE-1-0-G	NIMS-KMA/Republic of Korea
KIOST-ESM	KIOST/Republic of Korea
MIROC-ES2L	MIROC/Japan
MIROC6	MIROC/Japan
MPI-ESM1-2-HR	MPI-M, DWD, DKRZ/Germany
MPI-ESM1-2-LR	MPI-M, AWI, DKRZ, DWD/Germany
MRI-ESM2-0	MRI/Japan
NESM3	NUIST/China
NorESM2-LM	NCC/Norway
NorESM2-MM	NCC/Norway
UKESM1-0-LL	MOHC/UK

**Table 2:** Description of extreme temperature indices used in this study.

Index	Indicator name	Definition	Unit
TX90p	Warm days	Percentage of days when T_max_ > 90th percentile	%
TX10p	Cold days	Percentage of days when T_max_ < 10th percentile	%
TN90p	Warm nights	Percentage of days when T_min_ > 90th percentile	%
TN10p	Cold nights	Percentage of days when T_min_ < 10th percentile	%
HDs	Hot days	Annual count when T_max_ > 37.8 °C	Days
TR	Tropical nights	Annual count when daily T_min_ > 20 °C	Days
TXx	Maximum of T_max_	Annual maximum value of daily T_max_	°C
TNn	Minimum of T_min_	Annual minimum value of daily T_min_	°C

The CMIP6 model runs were developed to support the IPCC AR6. These datasets are recommended for assessing climate change impacts on processes sensitive to finer-scale gradients and the effects of local topography on climate conditions. In this study, we have adopted the GWL periods recommended by the IPCC AR6. These GWLs are classified as follows: 1.5 °C (2023–2042, 2021–2040, 2018–2037), 2 °C (never, 2043–2062, 2032–2051), 3 °C (never, never, 2055–2074), and 4 °C (never, never, 2075–2094) under the SSP1-2.6, SSP2-4.5, and SSP5-8.5 scenarios^1^. Additionally, to confirm these GWL periods for individual models, the starting and ending years of the relevant warming thresholds from CMIP6 (raw) models are provided in Table S1^35^. The selection of SSP1-2.6, SSP2-4.5, and SSP5-8.5 aimed to cover a spectrum of potential future trajectories, encompassing low, medium, and high-emission scenarios, respectively. In this study, we excluded the SSP3-7.0 scenario due to data insufficiency compared to the number of models available for other SSP scenarios. The IPCC Synthesis Report also mentioned that the GWLs are used to integrate the assessment of climate change and related impacts and risks since patterns of changes for many variables at a given GWLs are common to all scenarios considered and independent of timing when that level is reached^36^. This research delves into the implications of 1.5 °C, 2 °C, 3 °C, and 4 °C of GWLs across the AP, as countries grapple with diverse challenges stemming from climate change-induced extremes. The IPCC AR6 recently outlined the methodologies employed to determine the year when specific GWLs were first attained^1^, with similar approaches discussed in studies^37–39^. In conducting time series analysis, they adhere to the standard categorization of models based on their SSPs (SSP1-2.6, SSP2-4.5, and SSP5-8.5). When examining climatological averages, they adopt the framework of GWLs at 1.5 °C, 2 °C, 3 °C, and 4 °C rather than focusing on RCPs or SSPs at specific time intervals^40,41^. These GWLs are defined as the periods during which the 20-year running global average temperature reaches the corresponding level of change compared to the pre-industrial era of 1850–1900^42^. The present work adopts a set of common GWLs across which climate projections can be integrated. Here, the temperature extremes were calculated for the future period at GWLs of 1.5 °C, 2 °C, 3 °C, and 4 °C.

## Results

### Model evaluation

The annual cycle of T_max_, T_min_, and T_mean_ over the AP is shown from 1995 to 2014, using the MMM of NEX-GDDP-CMIP6 and ERA5 (Fig. S1a–c). The NEX-GDDP-CMIP6 MMM and ERA5 datasets showed similar patterns of monthly temperatures during the year, with higher values during the summer months (June to September) and lower values in the winter months (December to February). The magnitude of T_max_, T_min_, and T_mean_ are relatively less in the MMM than in the ERA5. The current study made use of the single realization of the selected climate models. Since we used the average of 27 models, to obtain a large MMM with their single realization, which also reduces the uncertainties in portraying the climate sensitivity, compared to each single model^43^. Furthermore, bringing all the selected climate models to uniform spatial resolution and integrating them into an MMM will certainly be helpful for the computational purpose and conventional evaluation of the models^44^. The annual mean values of T_max_, T_min_, and T_mean_ from the ERA5 and MMM are 32.7 (31.5) °C, 18.8 (19.0) °C, and 26.1 (25.6) °C respectively.

**Figure 1:**
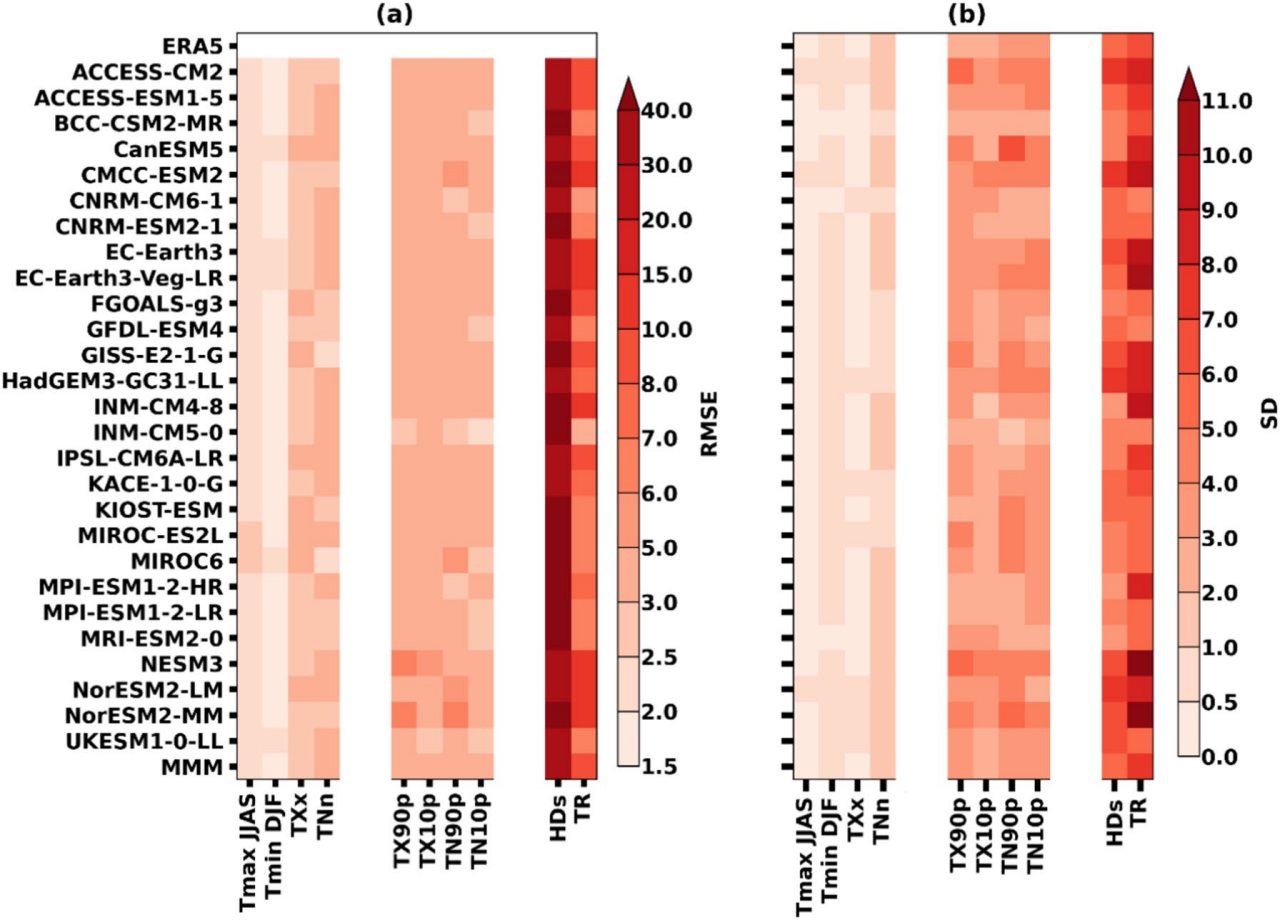
Performance of the NEX-GDDP-CMIP6 models in simulating historical temperatures and their extreme indices over the Arabian Peninsula (AP) region during the period 1995–2014; (**a**) Root mean square error (RMSE) and (**b**) standard deviation (SD), relative to the ERA5. The values are spatially averaged over AP for different climate indices of individual NEX-GDDP-CMIP6 models and multi-model mean (MMM).

Figure 1a,b show the root mean square (RMSE) and the standard deviation (SD) of all the individual models along with their NEX-GDDP-CMIP6 MMM and ERA5 for all the temperature extreme indices from 1995 to 2014. This analysis helps to understand the interannual variability and biases in the individual simulations when compared with the MMM and the reference dataset of ERA5. The RMSE and SD of MMM and ERA5 agree with varied differences in the respective models. Interestingly, the RMSE is relatively high (> 30) for HDs in all models, including MMM, and is followed by TR. Unlike the RMSE, the SD has shown higher values in most of the models, such as CMCC-ESM2, EC-Earth3, EC-Earth3-Veg-LR, INM-CM 4–8, NESM3, and NorESM-MM than the ERA5 and MMM in the case of TR. More SD is observed in TR, followed by HDs in all models, which can be attributed to their fixed threshold criteria. In such cases, it is difficult to capture the spatial relativity, when calculating the spatial average over a large area with different climatologies. The T_max_ of JJAS and T_min_ of DJF have shown good agreement with the ERA5 in all the model simulations, including MMM in the case of RMSE. The T_max_ of JJAS, T_min_ of DJF, and TXx showed a similarity in the magnitude of SD in all the models and MMM with the ERA5. Furthermore, a lesser difference is observed in the values of RMSE and SD of MMM in comparison with the ERA5.

### Future projections of temperature and extreme events

**Figure 2:**
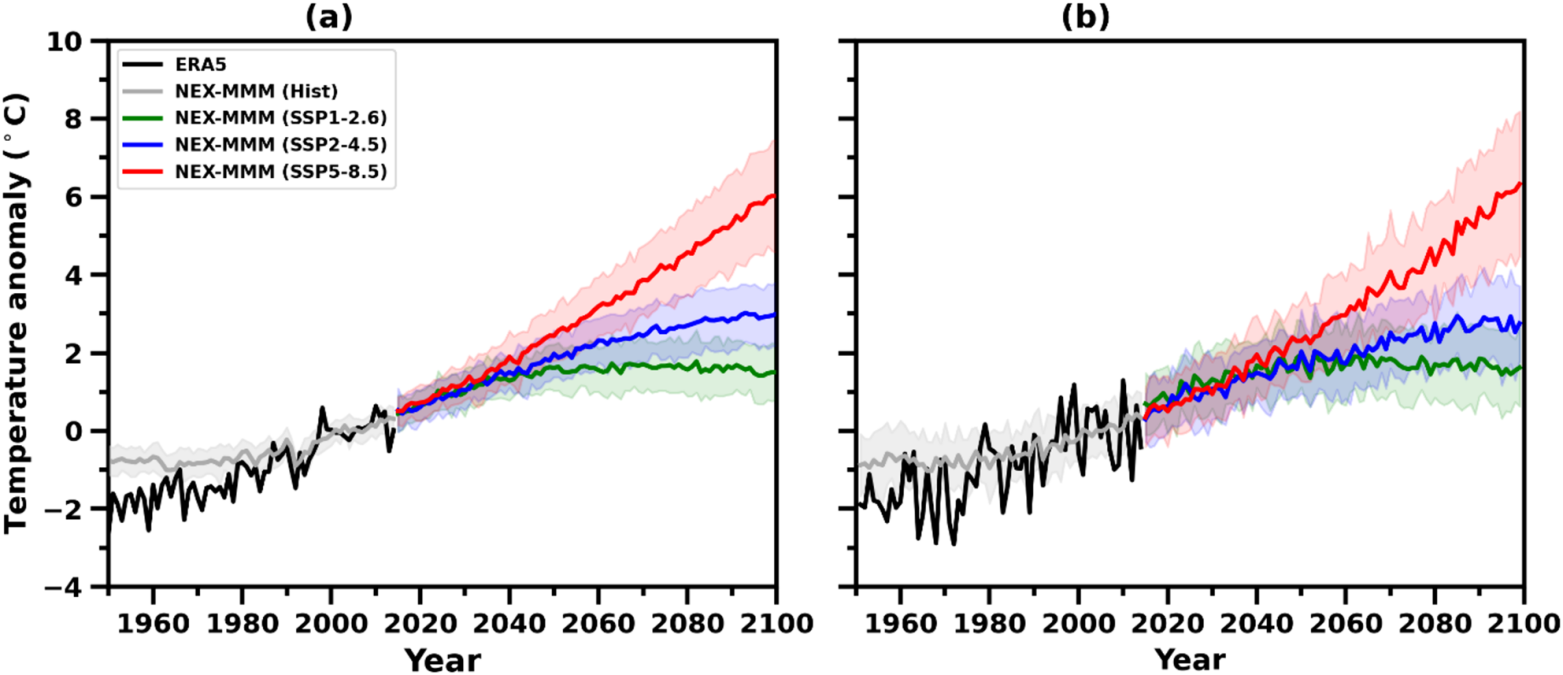
Historical (1951–2014) and projected (2015–2100) changes in annual anomalies of summer maximum temperature (T_max_) and winter minimum temperature (T_min_) from NEX-GDDP-CMIP6 MMM and ERA5 over the AP region under the selected SSPs, relative to the reference period (1995–2014); (**a**) summer T_max_ and (**b**) winter T_min_. Shading denotes the 27 NEX-GDDP-CMIP6 models' standard deviation.

Figure 2 displays the temporal evolution of the projected changes in summer T_max_ and winter T_min_ under SSP1-2.6, SSP2-4.5, and SSP5-8.5 scenarios. An increasing trend is observed in both T_max_ and T_min_. In particular, the warming is more pronounced from 2050 onward to the end of the twenty-first century in all the SSPs with varying degrees of warming. Relative to the reference period of 1995–2014, the high emission scenario (SSP5-8.5) has shown the maximum warming by yielding to + 6 °C in both the cases of T_max_ and T_min_. The summer T_max_ and winter T_min_ are projected to increase by 0.67 °C and 0.70 °C per decade respectively under SSP5-8.5. The medium (SSP2-4.5) and low (SSP1-2.6) scenarios showed a warming of + 2.9 °C and + 1.5 °C relative to the reference period. Projected increases of 0.11 °C (T_max_) and 0.09 °C (T_min_), as well as 0.31 °C (T_max_) and 0.28 °C (T_min_) per decade, are anticipated under the SSP1-2.6 and SSP2-4.5 scenarios respectively.

**Figure 3:**
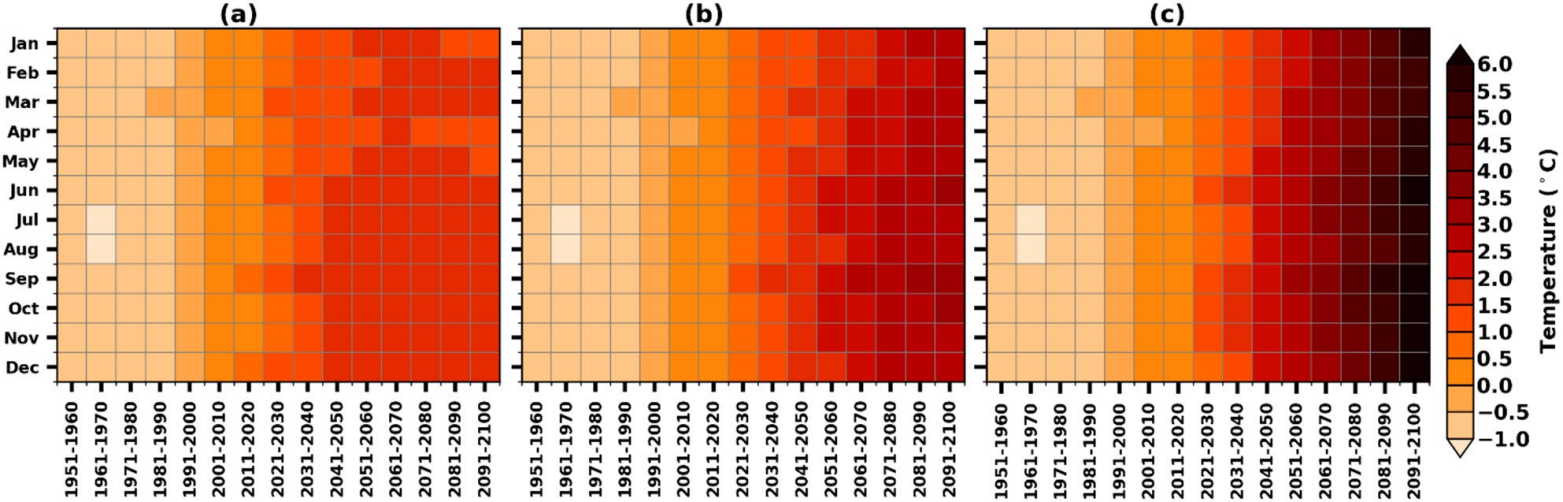
Historical and projected (1951–2100) decadal changes in monthly anomalies of mean temperatures from NEX-GDDP-CMIP6 MMM over the AP region under the selected SSPs, relative to the reference period (1995–2014); (**a**) SSP1-2.6, (**b**) SSP2-4.5, and (**c**) SSP5-8.5.

The monthly mean values of the temperatures for all the decades starting from 1951–1960 till 2091–2100 under the historical, SSP1-2.6, SSP2-4.5, and SSP5-8.5 scenarios are illustrated in Fig. 3. It is conspicuous that the monthly temperatures gradually increased from the decade of 1950s. This warming is more prominent in the SSP5-8.5 scenario than in the other two scenarios. It is clear from all the months, irrespective of the wet/dry season, that the warming is notable, up to 6 °C in most of the months and beyond 6 °C in June, September, October, and November months during the SSP5-8.5 scenario. A two-shift pattern has been observed in recording the elevated temperatures over the AP. The temperatures experienced a rise of 0.5 °C till the 1990s; thereafter the increase escalated to 1.6 °C, 3.0 °C and 6.0 °C under SSP1-2.6, 2–4.5, and 5–8.5 scenarios, respectively, toward the end of the century.

**Figure 4:**
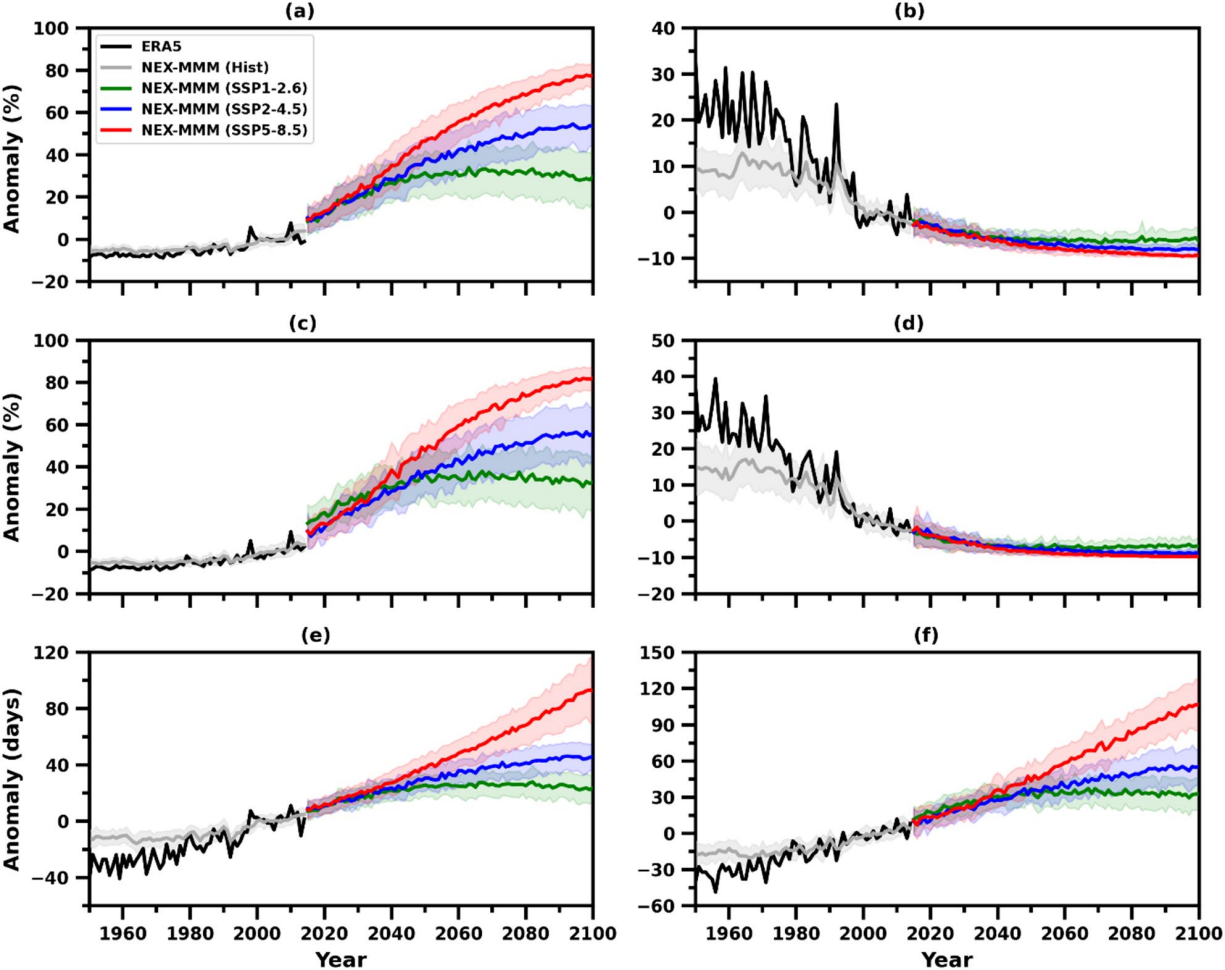
Historical (1951–2014) and projected (2015–2100) changes in annual anomalies of extreme temperature indices over the AP region from NEX-GDDP-CMIP6 MMM and ERA5 under the selected SSPs, relative to the reference period (1995–2014); (**a**) warm days (TX90p), (**b**) cold days (TX10p), (**c**) warm nights (TN90p), (**d**) cold nights (TN10p), (**e**) hot days (HDs), and (**f**) tropical nights (TR). Shading denotes the 27 NEX-GDDP-CMIP6 models' standard deviation.

An enormous amount of data indicates global warming has significantly increased the frequency and intensity of several climate extremes in the past few decades^8,45^. The annual temporal anomalies of temperature extremes over the AP region from MMM and ERA5 datasets for historical and future periods (1951–2100), relative to the reference period (1995–2014), are shown in Fig. 4. The results indicate that the daily temperature extremes, such as TX90p, TN90p, HDs, and TR, have shown an increasing trend in all SSP scenarios; however, SSP5-8.5 has a relatively higher magnitude of 77%, 82%, 90 days, and 106 days respectively, when compared with the other two scenarios by the year 2100. On the other hand, the daily cold temperature extremes, including the TX10p and TN10p, have shown a decreasing trend by the end of the twenty-first century in all SSP scenarios. The occurrence of TX10p and TN10p was between 20 to 30% during the 1950s, relative to the reference period; however, their magnitude has declined to − 10% by the end of the century. Figure 4 shows that the variability of ERA5 is higher than MMM in the temporal series of all temperature extremes during the historical period.

### Spatial variations of temperature extremes under different GWLs over AP

**Figure 5:**
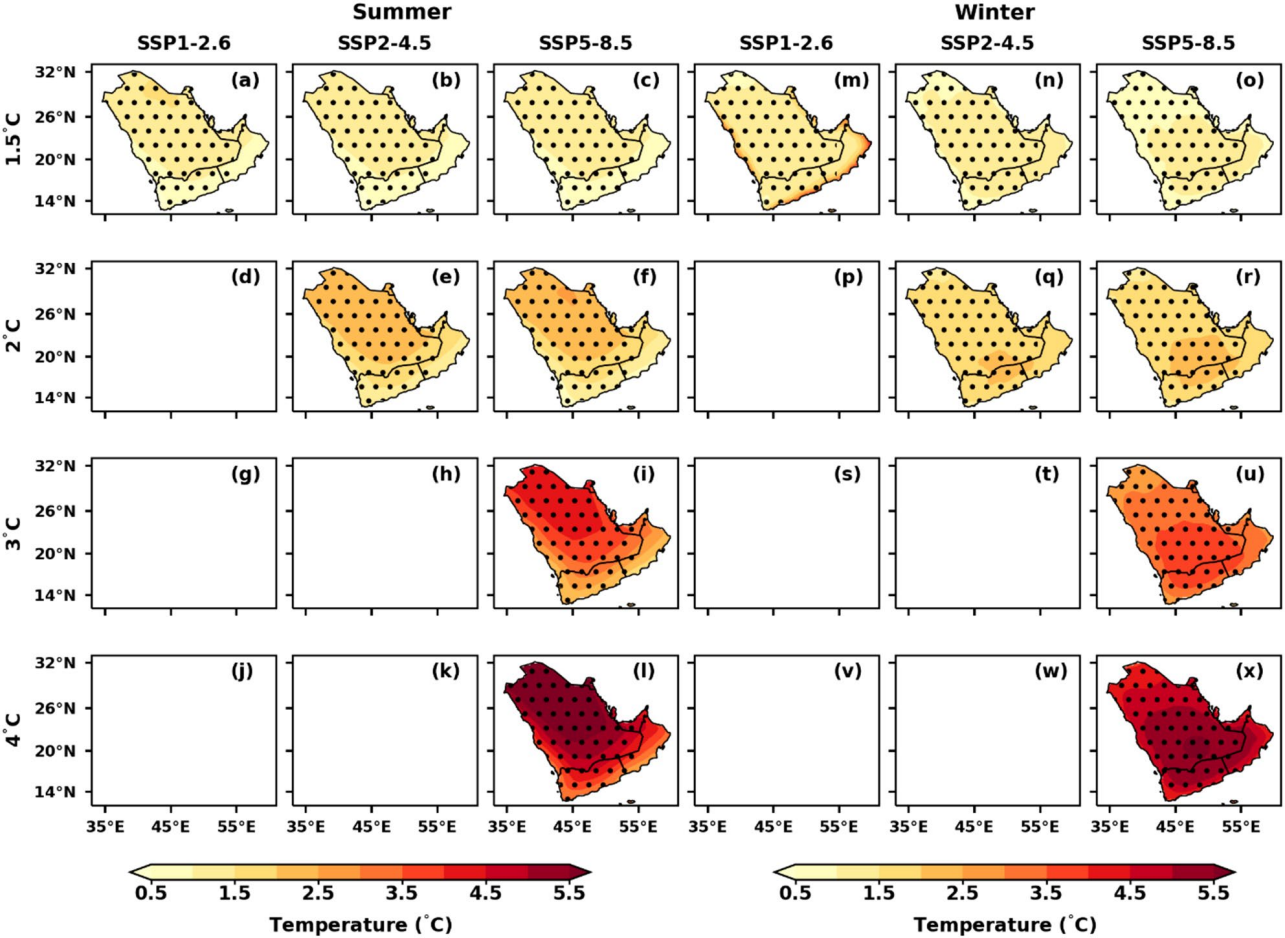
Spatial distribution of projected changes in summer T_max_ and winter T_min_ over the AP region under the selected GWLs and SSPs relative to the reference period (1995–2014); (**a**–**l**) summer T_min_ and (**m**–**x**) winter T_min_. Dotted regions represent significant changes at a 99% significance level.

**Figure 6:**
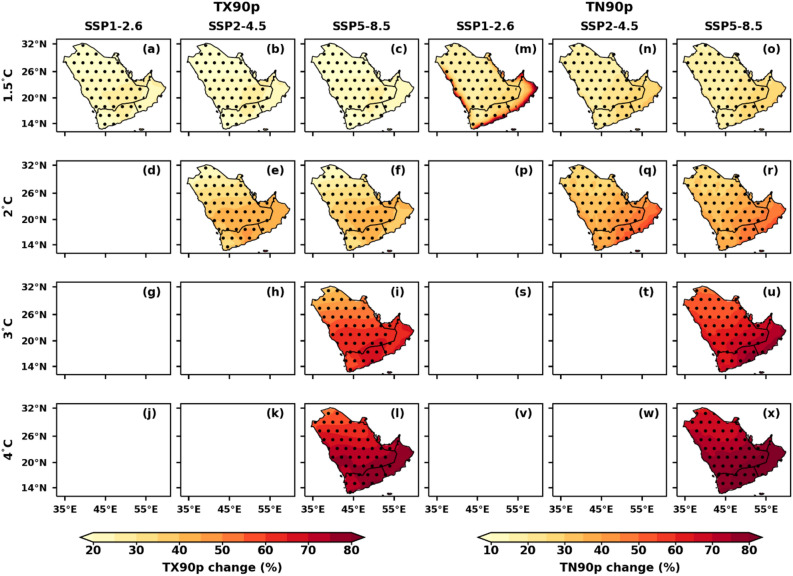
Spatial distribution of projected changes in annual warm days (TX90p) and warm nights (TN90p) over the AP region under the selected GWLs and SSPs relative to the reference period (1995–2014); (**a**–**l**) warm days (TX90p) and (**m**–**x**) warm nights (TN90p). Dotted regions represent significant changes at a 99% significance level.

**Figure 7:**
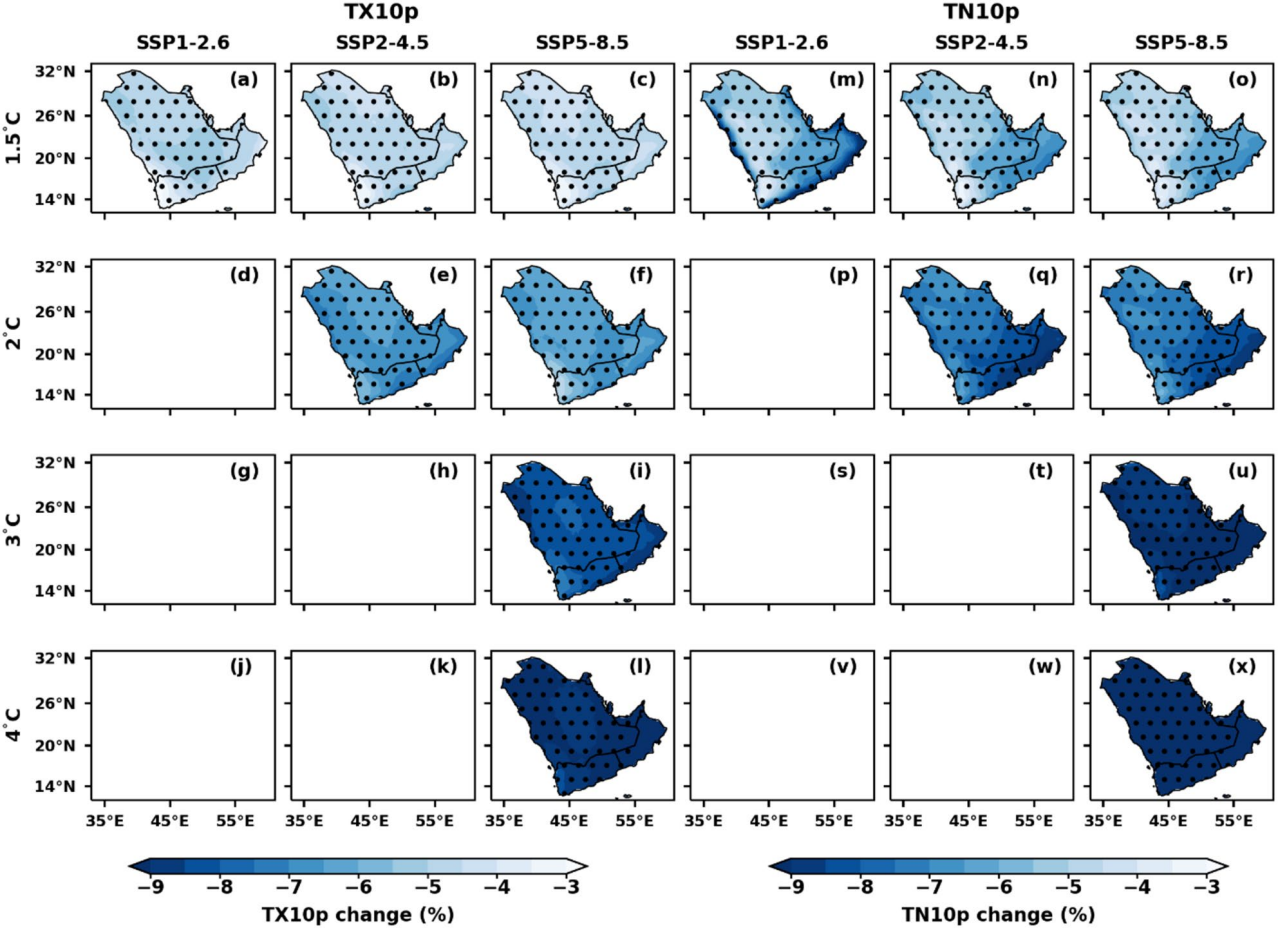
Spatial distribution of projected changes in annual cold days (TX10p) and cold nights (TN10p) over the AP region under the selected GWLs and SSPs, relative to the reference period (1995–2014); (**a**–**l**) cold days (TX10p) and (**m**–**x**) cold nights (TN10p). Dotted regions represent significant changes at a 99% significance level.

**Figure 8:**
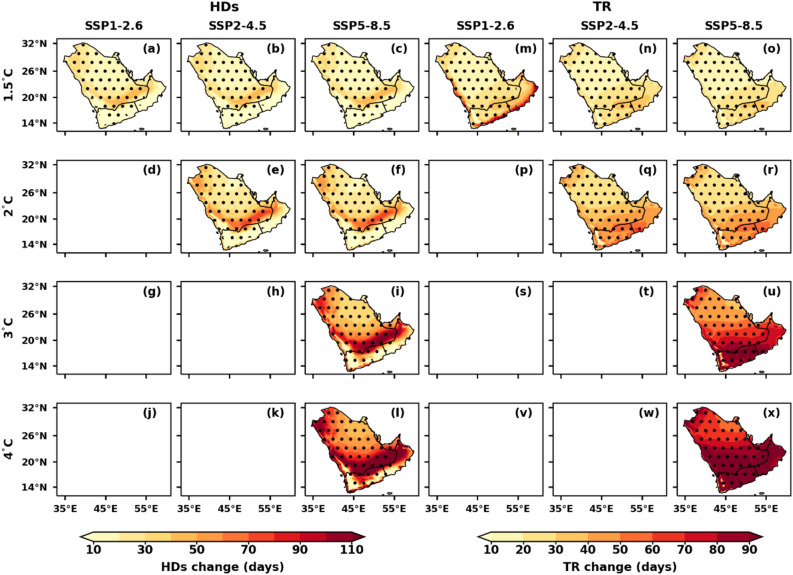
Spatial distribution of projected changes in annual hot days (HDs) and tropical nights (TR) over the AP region under the selected GWLs and SSPs, relative to the reference period (1995–2014); (**a**–**l**) hot days (HDs) and (**m**–**x**) tropical nights (TR). Dotted regions represent significant changes at a 99% significance level.

**Figure 9:**
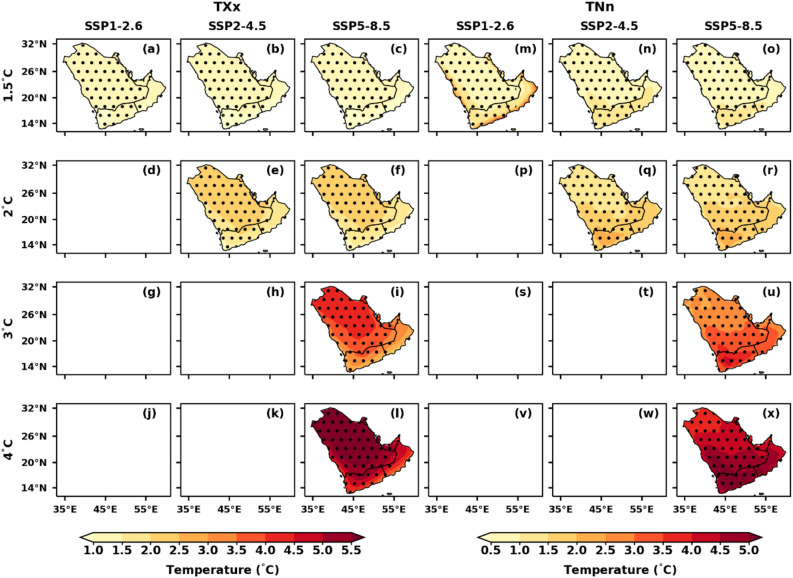
Spatial distribution of projected changes in annual maximum of T_max_ (TXx) and minimum of T_min_ (TNn) over the AP region under the selected GWLs and SSPs, relative to the reference period (1995–2014); (**a**–**l**) maximum of T_max_ (TXx) and (**m**–**x**) minimum of T_min_ (TNn). Dotted regions represent significant changes at a 99% significance level.

The projected relative changes in temperature extremes over the AP region under different GWLs and SSP scenarios are illustrated in Figs. 5, 6, 7, 8, 9. These projections, based on the reference period of 1995–2014, show relative changes in summer T_max_, winter T_min_, annual TX90p, TN90p, TX10p, TN10p, HDs, TR, TXx, and TNn under the 1.5 °C, 2 °C, 3 °C, and 4 °C GWLs for the SSP1-2.6, SSP2-4.5, and SSP5-8.5 scenarios.

The projected relative changes in summer T_max_, and winter T_min_ over the AP for the SSP1-2.6, SSP2-4.5, and SSP5-8.5 under the 1.5 °C–4 °C GWLs are shown in Fig. 5. The summer T_max_ and winter T_min_ are increasing across the AP, with T_max_ warming greater towards the north and central parts of AP (Fig. 5a–l). These changes are more prominent in the northern AP (beyond 2.0 °C warming) than in the southern AP (less than 2.0 °C warming). However, Oman did not show any significant change, though a warming tendency was observed. This phenomenon could be seen in Oman and some parts of the United Arab Emirates (UAE), which have mountains as well as long coastal lines, this may be one of the reasons to offset the extreme temperatures. Under the scenario of 3 °C warming, substantial changes in SSP5-8.5 are projected with a rise in temperature beyond 3.5 °C in the northern parts of AP and less than 2.5 °C in the southern parts, particularly in Yemen and Oman. The dominance of northern parts of AP can be seen in simulating the summer T_max_ of more than 5.5 °C under SSP5-8.5 of 4 °C GWL. The rise in winter T_min_ was mainly concentrated over the central and southern parts of the AP (4 °C GWL ) and extended toward the northern parts of the AP SSP5-8.5 (Fig. 5m–x). The summer T_max_ over AP for SSP1-2.6, SS2-4.5, and SSP5-8.5 shows negligible differences at a 1.5 °C GWL (Table 3). The T_max_ is projected to reach 41.0 °C and 42.5 °C at 3 °C and 4 °C GWLs under SSP5-8.5, respectively. The projected winter T_min_ is 14.7 °C at a 2 °C GWL under SSP2-4.5 and SSP5-8.5, and 17.8 °C at a 4 °C GWL under SSP5-8.5 (Table 3).

**Table 3:** Area-averaged values of temperatures and their extreme temperature indices under different GWLs and SSPs over the AP region.

Indices	Hist	1.5 °C			2 °C			3 °C			4 °C		
		SSP1-2.6	SSP2-4.5	SSP5-8.5	SSP1-2.6	SSP2-4.5	SSP5-8.5	SSP1-2.6	SSP2-4.5	SSP5-8.5	SSP1-2.6	SSP2-4.5	SSP5-8.5
JJAS T_max_ (°C)	37.6	38.8	38.7	38.7	–	39.6	39.5	–	–	41.0	–	–	42.5
DJF T_min_ (°C)	12.9	14.3	14.0	13.9	–	14.7	14.7	–	–	16.3	–	–	17.8
TX90p (%)	9.8	30	30.8	30.2	–	47	45.8	–	–	68.5	–	–	80.8
TX10p (%)	9.8	5	5.1	5.2	–	3.1	3.4	–	–	1.5	–	–	0.7
TN90p (%)	9.8	36.7	30.9	30.3	–	47.8	47.3	–	–	72.7	–	–	86.3
TN10p (%)	9.8	3.7	4.2	4.4	–	2.1	2.2	–	–	0.6	–	–	0.2
HDs (Days)	76.6	95	94.4	94.0	–	107	106	–	–	129	–	–	151
TR (Days)	188	213	209	208	–	224	224	–	–	252	–	–	286
TXx (°C)	41.4	42.6	42.5	42.5	–	43.4	43.4	–	–	45.1	–	–	46.7
TNn (°C)	7.2	8.3	8.13	8.0	–	8.7	8.8	–	–	10.3	–	–	11.7

The intriguing shifts in the spatial distribution of annual TX90p and TN90p, as depicted in Fig. 6, parallel each other in their responses to global warming. The number of TX90p and TN90P increase more rapidly over the southern parts of AP than in the northern parts, with an increment of more than 80% in the SSP5-8.5 scenario under 4 °C warming. The southern parts of AP have an increase of up to 50 to 60% days of TN90p under this 3 °C warming (Fig. 6). Consistent with increasing temperature (Figs. 2, 3), TX90p and TN90p increase, whereas TX10p and TN10p decrease over AP (Fig. 7). The number of TX10p decreased in the 1.5 °C warming category of SSP5-8.5 from 3 to 6% over AP. The number of TN10p decreased from − 3 days to − 9% days in the 1.5 °C to 3 °C warming category of SSP5-8.5 across the entire AP with a 99% significance level. The changes in TX10p and TN10p decreased by more than − 9 and − 8% days, respectively, over the entire AP under the 4 °C warming category of SSP5-8.5. The TX90p and TN90p are projected to reach up to 80.8% and 86.3%, respectively, at a 4 °C GWL under SSP5-8.5 (Table 3). The TX10p and TN10p are expected to decrease by a maximum of 0.7% and 0.2%, respectively, at a 4 °C GWL under SSP5-8.5 compared to other GWLs (Table 3).

The annual HDs maximum (more than 100 days) changes in the southern part of Saudi Arabia under the 4 °C warming of the SSP5-8.5 (Fig. 8). TR and TN90p show a similar pattern, TR is more than 70 days (> 90 days) over the southern part of AP at 3 °C (4 °C) GWLs under SSP5-8.5 (Fig. 8m–x). The number of HDs and TR in AP is projected to increase by 129 days and 252 days, respectively, at a 3 °C GWL under the SSP5-8.5 scenario (Table 3). At a 4 °C GWL under the same scenario, the HDs and TR are expected to rise by 151 and 286 days, respectively (Table 3). Under various GWLs, the TXx and TNn gradually increase compared to the baseline period (Fig. 9). More significant magnitude variations exist in TXx and TNn throughout the AP. The spatial variation of TXx (TNn) changes indicates an increase in the north and central AP (southern) region compared to the southern (northern) parts of AP. However, the TXx has shown the highest warming of more than 4 °C under 3 °C over the northern parts of AP in the SSP5-8.5. Under the SSP2-4.5 and SSP5-8.5 scenarios at a 2 °C GWL, the TXx (TNn) is projected to increase up to 43.4 °C (~ 8.8 °C) (Table 3). At a 4 °C GWL, the TXx (TNn) is expected to rise to 46.7 °C (11.7 °C) under the SSP5-8.5 scenario (Table 3).

As the warming threshold increases, all temperature indicators in AP are expected to increase, with a notable shift anticipated by the end of the twenty-first century. At high temperatures, the features are more noticeable. The changes in 2 °C warming, all the extremes show significance in SSP2-4.5 and SSP5-8.5 scenarios; note that SSP1-2.6 is not crossing the 2 °C warming level. The overall analysis suggests that the spatial variation of temperature extremes considered in the present study is statistically significant in most of the parts of AP, with remarkable changes in a few indices over the southern regions. In contrast, the changes in the rest of the indices are substantial in the northern parts of the AP.

## Discussion

AP is a climate change-sensitive region and is continuously undergoing significant warming. Recent research studies indicated a rise in surface temperature over different parts of AP during the historical and future periods. For instance, Almazouri et al.^46^ analyzed the Climate Research Unit (CRU) data and revealed that Saudi Arabia experienced a temperature rise of 0.72 °C and 0.51 °C per decade in the dry and wet seasons from 1979–2009 respectively. The ground-based observations and reanalysis of temperatures also showed an increasing trend in mean annual temperature over the AP^7,34^.

Given this significant warming in the AP region, this study was designed to estimate future temperature changes and their extremes over the AP for four different GWLs and three SSP scenarios. The results revealed that the mean annual cycle of the monthly temperatures of MMM, along with the ERA5, provide better insights into the comparison of modeled data with the reanalysis data set over the study region, where the long-term in-situ observations are not fully compatible in comparison to gridded modeled data. ERA5 is a proven dataset over this region, showing that the long-term climate variability is appropriate, as Bawadekji et al.^47^ reported. Fig. S1 shows that the NEX–GDDP-CMIP6 MMM was slightly underestimated during the historical period compared to ERA5 data. Similar variability of the models' simulation has been observed with the bias-corrected models over India when analyzed with the MMM of 13 CMIP6 models^48^. The model simulation could underestimate the mean monthly temperatures obtained from the India Meteorological Department dataset.

The model evaluation of the present study is shown in Fig. 1, unraveling a lesser bias with the MMM of the NEX–GDDP-CMIP6. Almazroui et al.^10^ used the CMIP6 data from the 31 models to study the future changes in climate over the AP region. CMIP6 data inferred a higher climate variability over the study region when compared with the CMIP5 data. In this perspective, the present study is of more relevance because it uses bias-corrected and high-resolution downscaled data from the NEX-GDDP-CMIP6 project. In addition, using MMM helps reduce the uncertainties originating from the raw CMIP6 data^23^. As reported by Ajjur and Al-Ghamdi^28^, AP is one of the global change hotspots, and the results obtained from the temporal anomalies of summer T_max_ and winter T_min_ over the AP region in the present study witness the same as the warming is going beyond 5 °C under SSP5-8.5 scenario by the end of the twenty-first century. These trends have been supported by the different regional trends over AP, reported by several studies^10,13,14^.

Using extreme temperature indices under different GWLs in the AP region makes the difference between the present work and the existing literature. The projected effects of climate variables under various warnings, such as 1.5 °C, 2 °C, 3 °C, and 4 °C have been widely reported across different regions of the globe, including Africa^49^, China^50^, the Caribbean region^51^, and South Asia^52^. As mentioned earlier, there are few studies on projected changes in temperatures and their extremes over the AP region under different SSPs; these changes have not been discussed in terms of varying warming levels, such as 1.5 °C, 2 °C, 3 °C, and 4 °C. To address this limitation, the present study quantifies the future variability of temperatures and their extremes in the AP region under four GWLs and three SSP scenarios. The study findings revealed more warming in summer T_max_ (winter T_min_) and related extremes over AP’s northern (southern) parts. Similar results were reported by Almazrouri et al.^10^ in the near and far future periods. For instance, they projected that the northern parts of AP during the winter season will likely experience an increase of 4.1–5.8 °C, while the southern regions are expected to have lesser warming. Likewise, in the present study, we have found major warming over northern parts of AP but with higher values than reported by Almazrouri et al.^10^. Our analysis revealed more warming witnessed, which can be attributed to the use of bias-corrected and high-resolution model simulations. Further, the increment of most of the temperature extremes poses threats that need to be tackled, particularly in the 4 °C warming of the SSP5-8.5 scenario. The southeastern parts of the AP, particularly southern South Arabia, Yemen, Oman, Qatar, and the United Arab Emirates, are to be the major focus in the warming scenario context, as these regions showed prominent changes in temperature extremes in all the warming categories of SSP5-8.5.

## Conclusions

Determining the future locations and levels of risk to lives and livelihoods requires understanding projected climate change and its spatial heterogeneity. Policymakers must fulfill this prerequisite to develop more realistic strategies for future climate change adaptation and mitigation. In the present study, we employed the high-resolution, statistically downscaled, and bias-corrected NEX-GDDP-CMIP6 models to estimate future temperature changes and their extremes over the AP region for four GWLs and three SSPs. The results suggest the NEX-GDDP-CMIP6 models can reproduce the monthly annual cycle pattern compared with ERA5 data during the reference period. The NEX-GDDP-CMIP6 individual and MMM showed the reproducing characteristics of extreme temperature events over the AP region. The summer T_max_ (winter T_min_) is expected to increase by 0.11–0.67 °C (0.09–0.69 °C) per decade under the selected SSPs by the end of the twenty-first century. The highest increase in summer T_max_ (winter T_min_) is expected to be observed in the central and northern parts of AP (southern part of AP) by the end of the twenty-first century. The results further reveal spatial annual variations in the patterns and magnitudes of temperature extremes, with substantial differences between the selected GWLs and SSPs. For 1.5 °C–4 °C GWLs, the annual changes in high-temperature extremes such as TX90p, TN90p, HDs, TR, TXx, and TNn are significantly increasing over the southern and central-north of AP, while the TX10p and TN10p days are expected to decrease over AP. These geographically specific patterns are of policy relevance. Some parts of the AP region will experience significant climate warming with substantial impacts even if the Paris Agreement goal is achieved.

### Supplementary Information

Supplementary Information.

## Data Availability

The NEX-GDDP-CMIP6 daily temperatures data can be obtained from the NASA data archive (https://www.nccs.nasa.gov/services/data-collections/land-based-products/nex-gddp-cmip6), while the ERA5 temperature hourly data can be accessed at https://cds.climate.copernicus.eu.
