# Supplementary material for: Amplification of temperature extremes in Arabian Peninsula under warmer worlds
Source: Sci Rep. 2024 Jul 18;14:16604. doi: 10.1038/s41598-024-67514-8 (PMC11258267; doi:10.1038/s41598-024-67514-8)
Supplement: Supplementary file 1 — Supplementary Information. [file 41598_2024_67514_MOESM1_ESM.docx]

**Amplification of temperature extremes in Arabian Peninsula under warmer worlds**

Buri Vinodhkumar ^1, 2, 3^, Safi Ullah ^1, 2^, T. V. Lakshmi Kumar ^4^, Sami G. Al-Ghamdi ^1, 2*^

^1^ Biological and Environmental Science and Engineering Division, Environmental Science and Engineering Program, King Abdullah University of Science and Technology (KAUST), Thuwal 23955-6900, Saudi Arabia

^2^ KAUST Climate and Livability Initiative, King Abdullah University of Science and Technology (KAUST), Thuwal 23955-6900, Saudi Arabia

^3^ Department of Earth and Atmospheric Sciences, National Institute of Technology Rourkela,

Rourkela 769008, India

^4^ School of Environmental Sciences, Jawaharlal Nehru University, New Delhi 110067, India

Buri Vinodh Kumar (<https://orcid.org/0000-0002-3986-5277>)

Safi Ullah (<https://orcid.org/0000-0002-2328-8321>)

T. V. Lakshmi Kumar (<https://orcid.org/0000-0002-6191-7969>)

Sami G. Al-Ghamdi (<https://orcid.org/0000-0002-7416-5153>)

*Corresponding Author's Email: sami.alghamdi@kaust.edu.sa

**Supplementary information**


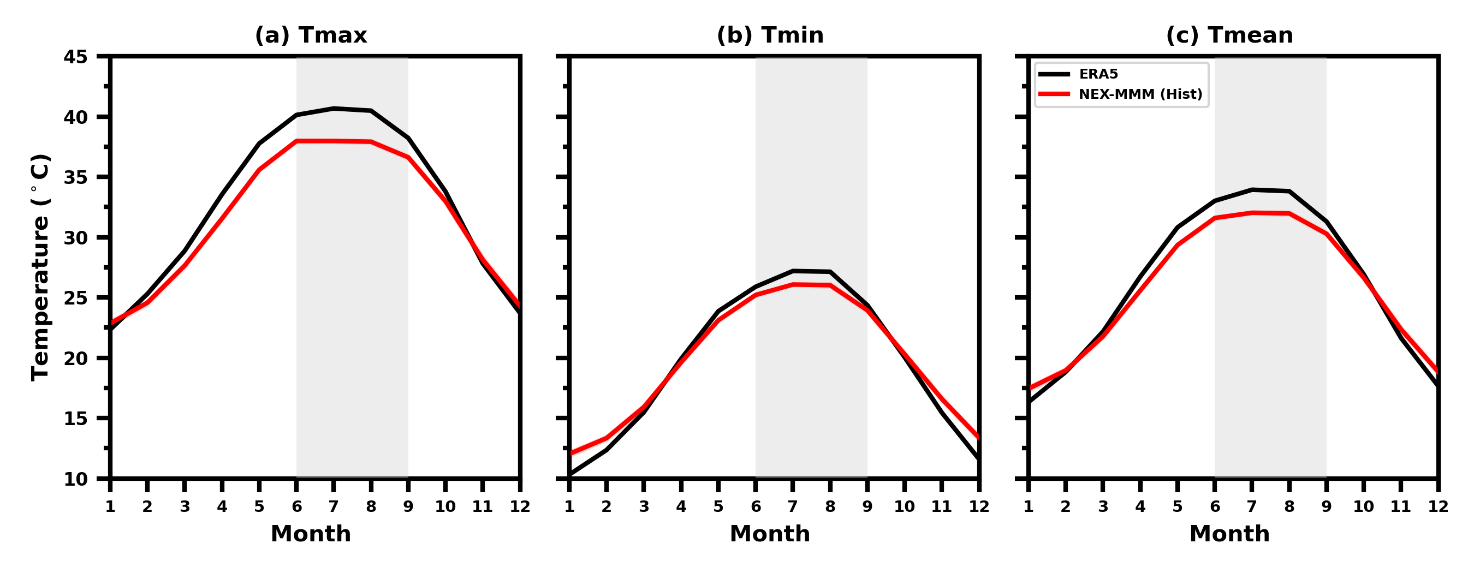


Figure S1. Annual cycle of temperatures over the Arabian Peninsula (AP) region from the NEX-GDDP-CMIP6 multi-model mean (MMM) during the period 1995–2014; (a) maximum temperature (T_max_), (b) minimum temperature (T_min_), and (c) mean (T_mean_) temperature. The gray-shaded region denotes the summer season (JJAS).

**Table S1.** CMIP6 individual (raw) models reach the relevant global warming thresholds above the pre-industrial level, under SSP1-2.6, SSP2-4.5, and SSP5-8.5 scenarios.

| **Model Name** | **1.5°C** | | | **2°C** | | | **3°C** | | | **4°C** | | |
| --- | --- | --- | --- | --- | --- | --- | --- | --- | --- | --- | --- | --- |
|  | **SSP1-2.6** | **SSP2-4.5** | **SSP5-8.5** | **SSP1-2.6** | **SSP2-4.5** | **SSP5-8.5** | **SSP1-2.6** | **SSP2-4.5** | **SSP5-8.5** | **SSP1-2.6** | **SSP2-4.5** | **SSP5-8.5** |
| ACCESS-CM2 |  |  |  |  |  |  |  |  |  |  |  |  |
| ACCESS-ESM1-5 |  |  |  |  |  |  |  |  |  |  |  |  |
| BCC-CSM2-MR | 2032–2051 | 2026–2045 | 2021–2040 |  | 2048–2067 | 2034–2053 |  |  | 2056–2075 |  |  |  |
| CanESM5 | 2004–2023 | 2004–2023 | 2003–2022 | 2017–2036 | 2015–2034 | 2013–2032 |  | 2040–2059 | 2031–2050 |  | 2074–2093 | 2045–2064 |
| CMCC-ESM2 |  |  |  |  |  |  |  |  |  |  |  |  |
| CNRM-CM6-1 | 2018–2037 | 2021–2040 | 2019–2038 |  |  |  |  |  |  |  |  |  |
| CNRM-ESM2-1 | 2038–2057 | 2028–2047 | 2023–2042 |  |  |  |  |  |  |  |  |  |
| EC-Earth3 | 2013–2032 | 2013–2032 | 2015–2034 | 2034–2053 | 2035–2054 | 2026–2045 |  | 2076–2095 | 2048–2067 |  |  | 2064–2083 |
| EC-Earth3-Veg-LR |  |  |  |  |  |  |  |  |  |  |  |  |
| FGOALS-g3 |  | 2022–2041 | 2020–2039 |  | 2056–2075 | 2038–2057 |  |  | 2065–2084 |  |  |  |
| GFDL-ESM4 |  | 2037–2056 | 2020–2039 |  | 2064–2083 | 2043–2062 |  |  | 2066–2085 |  |  |  |
| GISS-E2-1-G |  |  |  |  |  |  |  |  |  |  |  |  |
| HadGEM3-GC31-LL |  | 2010–2029 |  |  |  |  |  |  |  |  |  |  |
| INM-CM4-8 | 2041–2060 | 2026–2045 | 2021–2040 |  | 2054–2073 | 2037–2056 |  |  | 2060–2079 |  |  |  |
| INM-CM5-0 | 2027–2046 | 2028–2047 | 2021–2040 |  | 2063–2082 | 2037–2056 |  |  | 2065–2084 |  |  |  |
| IPSL-CM6A-LR | 2010–2029 | 2009–2028 | 2009–2028 | 2029–2048 | 2024–2043 | 2025–2044 |  | 2056–2075 | 2041–2060 |  |  | 2057–2076 |
| KACE-1-0-G |  |  |  |  |  |  |  |  |  |  |  |  |
| KIOST-ESM |  |  |  |  |  |  |  |  |  |  |  |  |
| MIROC-ES2L | 2032–2051 | 2032–2051 | 2025–2044 |  |  |  |  |  |  |  |  |  |
| MIROC6 | 2054–2073 | 2037–2056 | 2031–2050 |  | 2064–2083 | 2044–2063 |  |  | 2067–2086 |  |  |  |
| MPI-ESM1-2-HR | 2032–2051 | 2028–2047 | 2024–2043 |  | 2054–2073 | 2040–2059 |  |  | 2064–2083 |  |  |  |
| MPI-ESM1-2-LR |  |  |  |  |  |  |  |  |  |  |  |  |
| MRI-ESM2-0 | 2020–2039 | 2021–2040 | 2017–2036 |  | 2040–2059 | 2029–2048 |  |  | 2055–2074 |  |  | 2074–2093 |
| NESM3 | 2012–2031 | 2015–2034 | 2011–2030 | 2040–2059 | 2033–2052 | 2024–2043 |  |  | 2045–2064 |  |  | 2063–2082 |
| NorESM2-LM |  |  |  |  |  |  |  |  |  |  |  |  |
| NorESM2-MM |  |  |  |  |  |  |  |  |  |  |  |  |
| UKESM1-0-LL | 2014–2033 | 2014–2033 | 2014–2033 |  |  |  |  |  |  |  |  |  |
